# Case of Iodic Purpura

**Published:** 1878-05-20

**Authors:** Robert Abbe

**Affiliations:** New York


### CASE OF IODIC PURPURA.

By Robert Abbe, M.D., New York.

W. D., aged 30, England, in excellent general condition, presented himself with three deep tertiary ulcerations upon the anterior half of the tongue. He gives the history of having had primary and secondary syphilis about seven or eight years ago.

Six years ago he had swollen testicle, first on the right, then on the left side, which disappeared under the use of iodide of potassium, which did not then cause any eruption. A year and a half ago he had deep and painful ulcers of the back of the tongue which were cured by some medicine.

Four months ago, three ulcers came on the front portion of the tongue, which still remain; one the size of a pea, another the size of a three-cent piece, and another that of a penny. He was ordered at once, B. Potass. iodid, gr. x., Hydrag. biniodid. gr. 1-16, M. t. i. d.

The ulcers rapidly cicatrized, and were soon healed. After less than a fortnight's treatment an eruption of petechial spots appeared about the ankles and on the forearm above the wrists; those on the ankles were of deep purple color. There were not more than fifty or a hundred upon either ankle, while upon the forearms there were half that number. Upon stopping treatment as the tongue was cured, they disappeared. Shortly afterward, the medicine was again renewed and the eruption again appeared. It was continued only a few days when he seemed so well that no further observation was taken of the case until about three months after, Jan. 22, 1878, when the man presented himself with a very vivid and purpuric eruption of both legs and forearms, and with the following history:—Since he was last seen by me he had twice renewed the same medicine on account of not feeling particularly well, and after a few doses had both times had a vivid eruption appear as before; the time before the preseut one, he had taken but two doses (=pot. iod. gr. xx.) before it appeared, but it had quickly gone away on stopping the medicine.

On the present occasion he had taken the medicine, not because he had occasion to, but he had been out of work aud had nothing better to do. In two days the eruption was out in full force. It covered the entire anterior and posterior of the middle of the tribial region of the leg, and thinned ont toward the knee and about the ankle; few spots appearing below this point. In parts the spots were discrete, but for a space the size of one's hand spread out they merged together, giving the skin a deep hue.

On either forearm were very numerous purpuric spots pretty uniform in size, and extending from wrist to elbow; but none above elbow, or knee. The skin was otherwise normal; no elevation of temperature. There were no traces of *scorbutic* tendency; the gums were normal, and his general appearance was excellent. The patient had become alarmed, but was assured that all would disappear on stopping the medicine; and so it did with remarkable quickness, for in two weeks time not even the staining could be discovered.

P. S.—*March* 13. After taking three doses of the pot. iod. mixt. at my request, the purpuric eruption has again come out to-day, quite vividly on the legs and forearms. This is the sixth appearance of the eruption following the iodide. The fifth and sixth outbreaks of purpura were accompanied by sharp epistaxis.—*Dermatology.*

